# A double exponential potential for van der Waals interaction

**DOI:** 10.1063/1.5107505

**Published:** 2019-06-07

**Authors:** Xiongwu Wu, Bernard R. Brooks

**Affiliations:** Laboratory of Computational Biology, NHLBI, NIH, 12 South Dr., Bldg. 12A, Room 3053K, Bethesda, Maryland 20892, USA

## Abstract

Van der Waals (vdw) interaction is an important force between atoms and molecules. Many potential functions have been proposed to model vdw interaction such as the Lennard-Jones (L-J) potential. To overcome certain drawbacks of existing function forms, this work proposes a double exponential (DE) potential that contains a repulsive exponential term and an attractive exponential term. This potential decays faster than the L-J potential and has a soft core. The DE potential is very flexible and its two exponential parameters can be adjusted continuously to mimic many potential functions. Combined with the isotropic periodic sum (IPS) method, the DE potential can efficiently and accurately describe non-bonded interactions and is convenient for alchemical free energy calculation.

Molecular interactions determine macroscopic properties of matter. Potential functions are means for quantitative description of molecular interactions. Van der Waals (vdw) interaction is an important force between atoms and molecules. The Lennard-Jones (L-J) potential is a mathematically simple model that approximates vdw interaction. It was first proposed in 1924 by John Lennard-Jones.^1^

$$\varepsilon \left(r\right)=4\varepsilon_{0}\left({\left(\frac{\sigma }{\text{r}}\right)}^{12}-{\left(\frac{\sigma }{\text{r}}\right)}^{6}\right)=\varepsilon_{0}\left({\left(\frac{r_{m}}{\text{r}}\right)}^{12}-2{\left(\frac{r_{m}}{\text{r}}\right)}^{6}\right)$$ (1)

The function have a minimum of $-\varepsilon_{0}$ at $r=r_{m}$. σ is the distance where the potential is zero.

The L-J potential has only two parameters and is therefore limited in how accurately it can be fitted to the properties of any real material. The sixth-power term in the L-J potential models effectively the dipole–dipole interactions due to electron dispersion in noble gases (London dispersion forces), but it does not represent other kinds of interactions well. The twelfth-power term appearing in the potential is chosen for its ease of calculation for simulations (by squaring the sixth-power term) and is not theoretically or physically based.

Several other forms have been used to describe vdw interaction,^2^ such as the Buckingham potential:^3^

$$\varepsilon \left(r\right)=A\text{exp}\left(-Br\right)-\frac{C}{r^{6}}$$ (2)

which keeps the sixth-power term of the L-J potential but uses an exponential term to describe the repulsion. Study from the interacting quantum atoms (IQA) method has demonstrated that exponential relationships can better capture atomistic short-range repulsion.^4^

It has been found that the buffered 14−7 potential can better describe vdw interaction.^2^ The AMEOBA force field uses the buffered 14−7 potential and has found success in many applications.^5^

The L-J potential diverges when two atoms approach one another, which is also a problem for the $\frac{1}{r^{6}}$ term of the Buckingham potential. It is found the dispersion interaction can be better described with a damped $\frac{1}{r^{6}}$ term, indicating a new way to construct the dispersion component is needed for a new-generation force field.^6,7^

With above considerations, we propose a double exponential (DE) potential of the following form:

$$\varepsilon \left(r\right)=\varepsilon_{0}\left(\frac{\beta e^{\alpha }}{\alpha -\beta }\text{exp}\left(-\alpha \frac{r}{r_{m}}\right)-\frac{\alpha e^{\beta }}{\alpha -\beta }\text{exp}\left(-\beta \frac{r}{r_{m}}\right)\right)$$ (3)

The DE potential has a repulsive part in the form of $\text{exp}\left(-\alpha \frac{r}{r_{m}}\right)$ and an attraction part in the form of $\text{exp}\left(-\beta \frac{r}{r_{m}}\right)$. The exponential parameters, α and β, define the steepness of the repulsive interaction and the decay of the attraction, respectively. Like the L-J potential, the DE potential also has a minimum of $-\varepsilon_{0}$ at $r=r_{m}$.

The DE potential has many advantages. One advantage is that the DE potential has a soft core, $\varepsilon \left(0\right)=\varepsilon_{0}\left(\frac{\beta e^{\alpha }-\alpha e^{\beta }}{\alpha -\beta }\right)$, which make it convenient for free energy calculation through chemical perturbation. Another advantage of DE is that it converges faster than the L-J potential because the exponential functions decay faster than power functions. Third, the exponential parameters, α and β, can be adjusted continuously to mimic many potential functions. Table I lists the exponential parameters, α and β, that fit the DE potential to the $n_{a}-n_{c}$ potential ($n_{a}\in \left[2,14\right]\;and\;n_{c}\in \left[1,n_{a}-1\right]$) of the following form:^8^

$$\varepsilon \left(r\right)=\varepsilon_{0}\left(\frac{n_{c}}{n_{a}-n_{c}}{\left(\frac{r_{m}}{\text{r}}\right)}^{n_{a}}-\frac{n_{a}}{n_{a}-n_{c}}{\left(\frac{r_{m}}{\text{r}}\right)}^{n_{c}}\right)$$ (4)

When $n_{a}=12,n_{c}=6$ eq. (4) reduces to the L-J potential, eq. (1).

**TABLE I.** The DE parameters that best fit the DE potential, eq. (3), to the $n_{a}-n_{c}$ potential, eq. (4). The fitting is performed over potentials at $r/r_{m}\in \left(0.9,5.0\right)$ and with identical $\varepsilon_{0}$ and $r_{m}$.

$n_{a}$	$n_{c}$	a	c
2	1	19.745	0.285
3	1	22.226	0.352
3	2	16.941	0.683
4	1	23.657	0.395
4	2	17.600	0.805
4	3	14.071	1.194
5	1	24.621	0.424
5	2	18.337	0.896
5	3	14.401	1.371
5	4	12.978	1.797
6	1	25.346	0.445
6	2	19.015	0.965
6	3	14.987	1.509
6	4	13.413	2.014
6	5	13.045	2.455
7	1	25.939	0.460
7	2	19.635	1.019
7	3	15.648	1.619
7	4	14.028	2.188
7	5	13.576	2.699
7	6	13.672	3.146
8	1	26.458	0.471
8	2	20.218	1.062
8	3	16.331	1.709
8	4	14.726	2.330
8	5	14.238	2.900
8	6	14.283	3.409
8	7	14.613	3.856
9	1	26.934	0.481
9	2	20.784	1.097
9	3	17.027	1.782
9	4	15.475	2.447
9	5	14.982	3.066
9	6	14.994	3.629
9	7	15.277	4.133
9	8	15.722	4.579
10	1	27.389	0.488
10	2	21.349	1.125
10	3	17.739	1.843
10	4	16.262	2.544
10	5	15.782	3.204
10	6	15.774	3.813
10	7	16.016	4.367
10	8	16.409	4.866
10	9	16.906	5.312
11	1	27.837	0.494
11	2	21.945	1.149
11	3	18.473	1.893
11	4	17.083	2.625
11	5	16.626	3.319
11	6	16.603	3.968
11	7	16.809	4.566
11	8	17.155	5.113
11	9	17.599	5.609
11	10	18.119	6.055
12	1	28.288	0.499
12	2	22.520	1.170
12	3	19.235	1.936
12	4	17.935	2.693
12	5	17.504	3.416
12	6	17.470	4.099
12	7	17.644	4.736
12	8	17.946	5.326
12	9	18.341	5.867
12	10	18.810	6.361
12	11	19.341	6.808
13	1	28.749	0.503
13	2	23.141	1.187
13	3	20.027	1.972
13	4	18.817	2.750
13	5	18.410	3.500
13	6	18.365	4.210
13	7	18.510	4.881
13	8	18.774	5.509
13	9	19.124	6.092
13	10	19.545	6.630
13	11	20.027	7.122
13	12	20.564	7.570
14	1	29.227	0.507
14	2	23.793	1.202
14	3	20.851	2.003
14	4	19.724	2.799
14	5	19.339	3.569
14	6	19.283	4.306
14	7	19.401	5.006
14	8	19.630	5.667
14	9	19.940	6.287
14	10	20.317	6.865
14	11	20.754	7.399
14	12	21.244	7.891
14	13	21.785	8.339

For efficient calculation of long-range nonbonded interactions, we have developed the isotropic periodic sum (IPS) method.^9^ For a power function, $\frac{1}{r^{n}}$, a simple IPS potential in the form of $r^{m}$ have been derived based on the homogeneity condition:^8^

$$\phi_{\text{p}}^{\text{IPS}}\left(r,r_{c},n,m\right)=\frac{n}{m}\frac{r^{m}}{r_{c}^{n+m}}+\left(\frac{n}{\left(m+3\right)\left(n-3\right)}-\frac{1}{m}\right)\frac{m+n}{r_{c}^{n}}$$ (5)

where $r_{c}$ is the radius of the IPS local region, which is also called the cutoff distance. The IPS potential power *m* can be any integer. We pick m=10 and 4 for n=12 and 6, respectively, because they make eq. (5) best fit the analytically solved IPS potentials.^8^ Therefore, the L-J IPS potential has the following form:

$$\varepsilon_{\text{LJ}}^{\text{IPS}}\left(r,r_{c}\right)=\left\{\begin{array}{cc} \varepsilon_{0}\left(r_{m}^{12}\left(\frac{1}{{\text{r}}^{12}}+\phi_{\text{p}}^{\text{IPS}}\left(r,r_{c},12,10\right)\right)-2r_{m}^{6}\left(\frac{1}{{\text{r}}^{6}}+\phi_{\text{p}}^{\text{IPS}}\left(r,r_{c},6,4\right)\right)\right) & r\leq r_{c}\\ 0 & r>r_{c} \end{array}\right.$$ (6)

Similarly, to efficiently handle long-range DE interaction, we can derive a simple IPS potential for the exponential function, $e^{-\kappa \text{r}}$, based on the homogeneity condition:^8^

$$\phi_{\text{E}}^{\text{IPS}}\left(r,r_{c},\kappa \right)=e^{\kappa \left(r-2r_{c}\right)}+\frac{12e^{-\kappa r_{c}}}{\kappa^{2}r_{c}^{2}}+\frac{6e^{-2\kappa r_{c}}}{\kappa^{3}r_{c}^{3}}$$ (7)

The DE IPS potential has the following form:

$$\varepsilon_{\text{DE}}^{\text{IPS}}\left(r,r_{c}\right)=\left\{\begin{array}{cc} \varepsilon_{0}\left(\frac{\beta e^{\alpha }}{\alpha -\beta }\left(\text{exp}\left(-\alpha \frac{r}{r_{m}}\right)+\phi_{\text{E}}^{\text{IPS}}\left(r,r_{c},\frac{\alpha }{r_{m}}\right)\right)-\frac{\alpha e^{\beta }}{\alpha -\beta }\left(\text{exp}\left(-\beta \frac{r}{r_{m}}\right)+\phi_{\text{E}}^{\text{IPS}}\left(r,r_{c},\frac{\beta }{r_{m}}\right)\right)\right) & r\leq r_{c}\\ 0 & r>r_{c} \end{array}\right.$$ (8)

One major concern about the DE potential, eq. (3), is the expensive calculation of the two exponential functions, $\text{exp}\left(-\alpha \frac{r}{r_{m}}\right)$ and $\text{exp}\left(-\beta \frac{r}{r_{m}}\right)$. This burden has been greatly reduced by the fast approximation functions proposed to replace exponential function calculation.^10^ On GPU, CUDA’s implementation of the single-precision exponential function, expf(), makes use of the fast hardware approximation and significantly speed up the calculation. The DE IPS potential, eq. (8), does not increase the calculation cost very much as compared with the DE potential, eq. (3), because the first exponential function in eq. (7) is related to a reciprocal of the exponential function in the DE potential and the other two exponential functions in eq. (7) are constants that can be precalculated. Therefore, the DE IPS potential, eq. (8), has only two exponentials to calculate for each pair of particles, the same as the DE potential, eq. (3).

The DE potential can mimic many types of repulsive-attractive potentials like Lennard-Jones potential. As shown in Table I, the DE potential that mimic the L-J potentials has α=17.470 and β=4.099. For convenience, we use DE (α, β) to represent a DE potential with the given α, β values. Fig. 1 (bottom) shows the L-J potential, DE (17.470, 4.099) and DE (1.1, 1.0). Clearly, DE (17.470, 4.099) overlaps with L-J potential very well around the well region. The potential DE (1.1, 1.0) shows an flat energy surface with an accessible core region around *r*=0. Small α, β values result in flat energy surface, which corresponds to a slow decay, and a large cutoff distance would be needed for the cutoff method to have converged ensemble averages. To avoid large cutoff distances, one can use the IPS method to obtain converged results with reasonable small cutoff distances. The middle panel of Fig. 1 shows their corresponding IPS potentials, eq. (6) and eq. (8), which are short ranged within the cutoff distance, here, $r_{c}=3r_{m}$. The top panel shows the IPS pair potentials, $\varepsilon_{\text{pair}}^{\text{IPS}}\left(r,r_{c}\right)=\varepsilon^{\text{IPS}}\left(r,r_{c}\right)-\varepsilon^{\text{IPS}}\left(r_{c},r_{c}\right)$, which are continuous and smooth at the cutoff boundary.

**FIG. 1.**
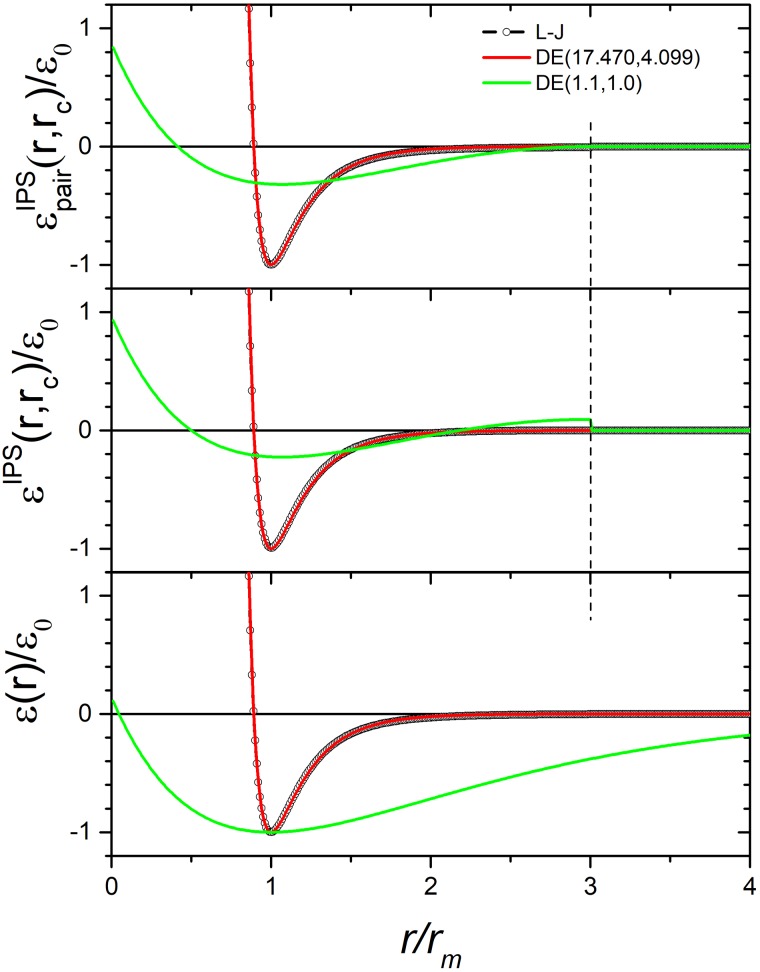
The Lennard-Jones potential and the double exponential potentials. Bottom panel compares the L-J potential with DE (17.470,4.099) and DE (1.1, 1.0). Middle panel compares their IPS potentials. Topper panel compares their IPS pair potentials. The cutoff distance for the IPS potentials is $r_{c}=3r_{m}$.

To illustrate the application of the DE potential, a cubic box of 500 particles are simulated with $\varepsilon_{0}=119.8K=0.238kcal/mol$ and $r_{m}=3.822$Å, which are used as the Lennard-Jones parameters for argon. The cubic box side is 28.53Å and the periodic boundary condition (PBC) is applied. 10ns *NVT* molecular dynamics simulations are performed for each data point. Cutoff distances of 6, 8, 10, 12, 15, and 20Å are used to examine simulation convergency. Simulations with a straight cutoff method where energy goes to zero when $r>r_{c}$ are performed to compare with the IPS method.

*NVT* simulations are performed at *T*=100K for the L-J and the DE (17.470, 4.099) systems, and *T=*10^5^ K for the DE (1.1, 1.0) systems to keep the systems in a fluid state. The average potential energies at different cutoff distances are shown in Fig. 2. Clearly, the average energies of the three systems are different. Even though the DE (17.470, 4.099) potential mimics the L-J potential, they are different in many ways: core softness, long-range decay, and IPS potentials. They are similar around the well and have exactly the same well depth, $-\varepsilon_{0}$, and well location, $r_{m}$ (see Fig. 1), but the mentioned differences will result in different average energies. Therefore, to use the DE potential for vdw interaction, the parameters, $\varepsilon_{0}$ and $r_{m}$, need be reparametrized and should not be taken directly from the L-J parameters.

**FIG. 2.**
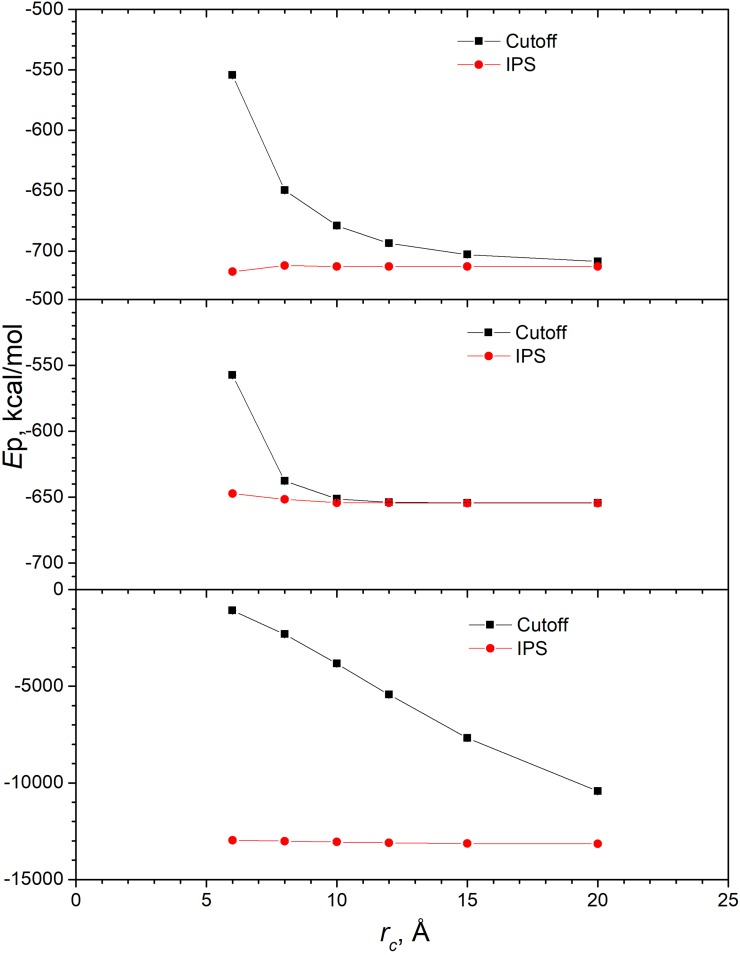
Average potential energies of the L-J system and the DE systems from *NVT* simulations at different cutoff distances. Top panel: the Lennard-Jones system. Middle panel: the DE (17.470, 4.099) system. Bottom panel: the DE (1.1,1.0) system.

Comparing the cutoff and the IPS results we can see clearly the IPS method produces converged results with a cutoff distance as small as 8 Å. The cutoff method needs much larger cutoff distance to reach the converged result. For the DE (17.470, 4.099) system, the cutoff results reach the IPS converged result at 12 Å (Fig. 2, middle panel), while for the L-J system the cutoff results do not reach the IPS converged result until 20 Å (Fig. 2, top panel), demonstrating that the DE potential decays faster than the L-J potential.

When α, β are small, the potential decay is slow and larger cutoff distances are needed for the cutoff method to obtain converged results. As can be seen from the bottom panel of Fig. 2, for the DE (1.1,1.0) system, even with a cutoff distance of 20 Å, the cutoff result is still far away from the IPS converged value. Again, the IPS results is almost independent of the cutoff distance. Therefore, for DE with small α, β values, the IPS method has significant advantage over the cutoff method.

The soft core of the DE potential can be clearly seen from the radial distribution functions (RDF) shown in Fig. 3. The RDF of the L-J system and the DE (17.470, 4.099) system are almost identical, indicating the DE potential is a good mimic of the L-J potential. For the DE (1.1, 1.0) system, we can see all distance become accessible, indicating there is no hard core to stop particles from approaching. This is an important advantage of the DE potential that makes it suitable for alchemical free energy calculation.

**FIG. 3.**
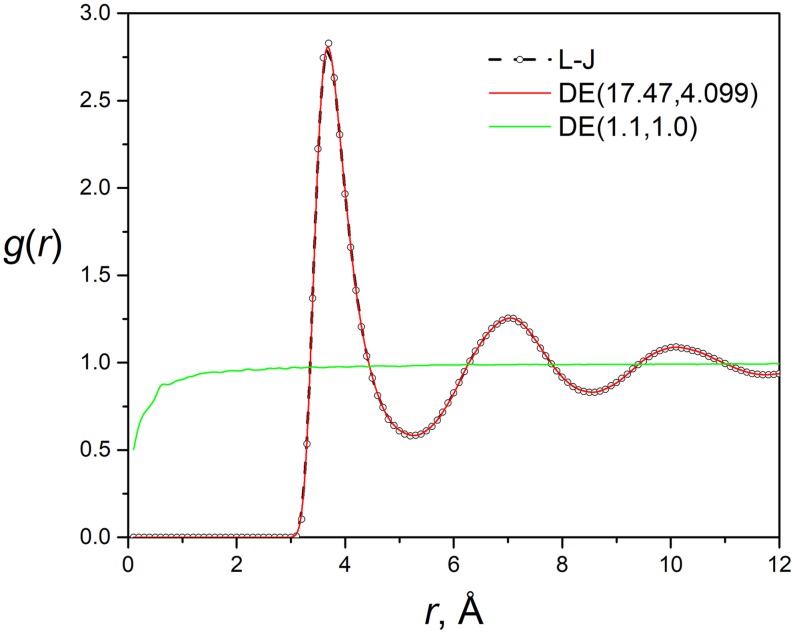
Radial distribution functions of the L-J system and the DE systems from *NVT* simulations.

With the four parameters, α, β, $\varepsilon_{0}$, and $r_{m}$, the DE potential is flexible enough to better fit into experiment properties of real matter. We will examine the DE potential in term of parameter fitting and property prediction in our future work. With all its advantages, we believe that the DE potential will find roles in new force field development and in molecular simulation.
